# Effect of Initial Surface Morphology and Laser Parameters on the Laser Polishing of Stainless Steel Manufactured by Laser Powder Bed Fusion

**DOI:** 10.3390/ma17204968

**Published:** 2024-10-11

**Authors:** Jiangwei Liu, Kangkang Zhao, Xiebin Wang, Hu Li

**Affiliations:** 1 School of Energy and Power Engineering, Shandong University, Jinan 250061, China; 2 Key Laboratory for Liquid-Solid Structural Evolution and Processing of Materials (Ministry of Education), Shandong University, Jinan 250061, China; 3 Shandong Technology Centre of Nanodevices and Integration, School of Integrated Circuits, Shandong University, Jinan 250101, China; 4 Shenzhen Research Institute of Shandong University, Shenzhen 518000, China; 5 Department of Materials Science and Engineering, Uppsala University, 75121 Uppsala, Sweden

**Keywords:** laser powder bed fusion, surface roughness, laser polishing, response surface methodology, process optimization

## Abstract

The topological characteristics of the down-skin surfaces for as-built components by laser powder bed fusion (LPBF) are particularly representative, while the study on the improvement of the surface quality of these surfaces remains largely unexplored. Herein, the laser polishing of LPBF-built components with different inclination angles was systematically investigated with an emphasis on the down-skin surfaces. Our result shows that the topography of the top surface is independent of the inclination angle, and the surface topography of the down-skin surface is dominated by additional angle-dependent surface characteristics. It also indicates that the surface roughness can be reduced sharply when increasing the laser power from 40 W to 60 W, and the reduction slows down when further increasing the laser power while decreasing the scanning speed leads to a progressive improvement of the surface morphology. Moreover, a second-order regression model was established to evaluate the influence of the initial surface morphology and polishing parameters on the polished surface roughness and to achieve surface roughness optimization. Therefore, our established methodology can be readily applied to surface morphology manipulation and process optimization for laser polishing of widely used metals and alloys fabricated by the additive manufacturing process.

## 1. Introduction

Additive Manufacturing (AM) opens up numerous opportunities for the manufacturing of complex parts and new possibilities for innovative part design [1]. Even though the AM process can provide fine geometry precision [2], excellent mechanical properties [3], as well as the prospect of achieving functionalized structures of manufactured components [4], the surface topography of AM-built components is still a key limitation factor, which is attracting considerable attention [5]. In situ improvement has been carried out, and the effect of the energy input on the surface roughness during the laser powder bed fusion (LPBF) has been studied [6,7,8]. Yuan et al. [7] discovered that varying the laser scanning speed may achieve three states of the molten pools: unstable state, transition state, and stable state. It is reported that sufficient energy input guarantees a smooth melt surface, while low laser power results in an unstable flow of the molten pool [9]. A suitable hatching distance is also found to be necessary for manufacturing a smooth surface [10], and the distortions and breakups of the molten tracks are strongly associated with the powder distribution [11]. To quantify the interdependency of the build/inclination angle between the manufactured part and the laser incidence, the surface laser relation angle was introduced, and the results revealed that the surface roughness is considerably decreased by employing a suitable surface laser relation angle [12]. Existing attempts at in situ improvement mainly aim at the morphology improvement on the top surfaces of LPBF-built samples, while it is still difficult to eliminate the adhered powder particles on the side surfaces. To provide components acceptable for engineering applications, desirable surface roughness is compulsory. Therefore, additional post-treatments are normally compulsory to enhance the surface quality of the LPBF-built components [13].

During the past several decades, laser polishing has demonstrated continuous development in the capability of reducing the surface roughness of a series of materials such as ceramics, metals, and polymers [14]. Unlike mechanical surface treatment, laser polishing employs thermal energy to reduce the surface roughness by ablating or melting a small amount of materials. By employing a continuous wave laser, macrostructural smoothing can be achieved with the mechanism of melting and swift solidification of the surface layer. Depending upon the depth of the melted layer, the mechanism of laser polishing consists of two types: surface shallow melting (SSM) and surface over melting (SOM) [15]. In SSM, the surface roughness is reduced by melting the surface peaks and filling the valleys. When the laser intensity increases further, the depth of the melted layer may go beyond the valleys, and the onset of SOM can approach. In SOM, surface waves with high amplitude but low frequency can lead to an increased surface roughness [16]. Therefore, the identification of the polishing regime is dictated by both the energy density during polishing and the initial surface status prior to laser polishing, which should be evaluated simultaneously when determining the polishing mechanism [17].

For the as-built surfaces of LPBF-built components, the side surfaces normally have higher surface roughness, which is inherent to the layer-wise process [18]. The side surfaces can be classified into two categories: the down-skin (the downward-facing) surface and the up-skin (the upward-facing) surface. It is found that the surface roughness of up-skin surfaces is 2–17 µm, while the roughness value of the down-skin surfaces is over 25 µm [19]. For the typical AM-manufactured components, the area of top surfaces is ignorable compared with the high proportion of the up-skin and down-skin areas of the manufactured components [20,21]. Current research focuses mainly on the top surfaces [22,23] or the side surfaces of the vertically fabricated specimen [24,25]. For instance, Narayanan et al. [19] performed the surface polishing of LPBF-built samples, where the up-skin surface with different inclination angles was considered. However, the situation of the surface morphology on the down-skin surfaces is quite different, which not only relies on the geometry of the step edge and the adhesion of the partially melted metal particles but is also highly related to the infiltration of the melt pool into the powder bed [26]. The topological characteristics of the down-skin surface are particularly representative of AM-built components compared with those of the up-skin surface and the top surface of LPBF-built specimens. It is, therefore, of great importance to conduct investigations on the laser polishing of the down-skin surfaces of the LPBF-built component.

Machine learning approaches such as response surface methodology [27], gaussian process regression [28], and backpropagation (BP) neural networks [29] have been proven to be more effective and applicable approaches compared to experimental methods when performing the process optimization of AM-built components. Among those approaches, the response surface methodology is a unique approach, which expresses the effect of numerous key factors on the response by establishing a polynomial and finding an optimal solution for factors with exceptional responses. One prominent advantage of this method is to give a mathematical expression not only expressing the correlation between the factors and responses but also containing the interaction between factors [27]. Therefore, it has been widely employed as an efficient approach for the parameter optimization of the LPBF process [30].

Considering the fact that the complexity and roughness of the down-skin surface are more representative than the up-skin surface, the down-skin surface of LPBF-built samples is positioned as the target to perform laser polishing in this work. The effect of the initial surface features as well as the polishing parameters on the final polished morphology was therefore evaluated, and a second-order regression model based on process parameters was then established for the optimization of the surface roughness of laser polishing.

## 2. Materials and Methods

### 2.1. Sample Preparations

**Figure 1:**
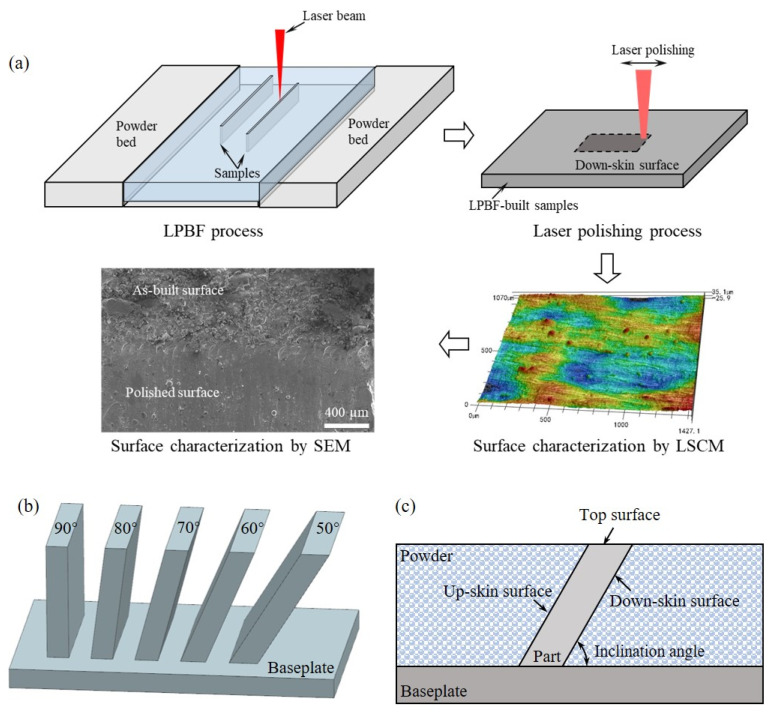
(**a**) Flowchart of the steps of the experimental process. Schematic view of (**b**) the inclination angles varying from 50° to 90° and (**c**) the surfaces on a LPBF-built specimen.

The sample preparation and experimental steps are shown in Figure 1a. The feedstock material for the LPBF process was 316L stainless steel powder (LaserForm 316L (A), 3D Systems, USA) with a mean diameter of 21.9 μm. A ProX DMP 320 LPBF instrument (3D Systems) equipped with a 500 W fiber laser (1070 nm in wavelength) was employed to fabricate the as-built samples. Before starting the LPBF process, ultra-pure argon was used to maintain a low oxygen concentration (<25 ppm) during the LPBF process. Processing parameters are summarized in Table 1 [3]. In order to evaluate the influence of surface morphology, the inclination angles of LPBF-built samples were varied from 50° to 90° considering the limitation of self-supporting [31], as shown in Figure 1b. Side surfaces and top surfaces of the LPBF-built specimens were characterized in this work. The side surface mentioned in this work denotes the down-skin surface of the specimen, as shown in Figure 1c.

### 2.2. Laser Polishing Process

After LPBF fabrication, the as-built samples were cut from the baseplate by using a wire electrical discharge machine. Laser polishing was performed by employing the fiber laser equipped within a ProX DMP 320 machine (3D Systems, USA). For each polishing case, the size of the processed area was 10 × 10 mm^2^ on the down-skin surface of LPBF-built samples (indicated in Figure 1b). The detailed polishing parameters are illustrated in Table 2, and a line step-over distance of 0.04 mm was used to maintain a sufficient overlap during laser polishing. Energy density *E* (J/mm^2^) is calculated using the following relation:

(1) E=P/(V×D)

where *P*, *V*, and *D* are the laser power in watts, the scanning speed in mm/s, and the laser spot diameter in μm, respectively. For each condition in Table 2, three measurements were performed, and the average value was used in this paper.

### 2.3. Characterization Methods

A 3D laser scanning confocal microscope (VK-X200K, Keyence, Osaka, Japan) was employed to characterize the 3D surface profile of as-built and laser polished samples. Based on the ISO 25178-2 standard [32], the height parameters, including the areal roughness (*S_a_*), profile roughness (*R_a_*), and kurtosis (*S_ku_*), and the features parameters, encompassing the density of peaks (*S_pd_*), the maximum valley depth (*S_v_*), and the maximum peak height (*S_p_*), were used to quantitatively evaluate the surface morphology in this paper. A scanning electron microscope (SEM) was employed to image the surface of LPBF-built and laser-polished specimens.

## 3. Experimental Results

### 3.1. Surface Characterizations of the LPBF-Built Specimens

**Figure 2:**
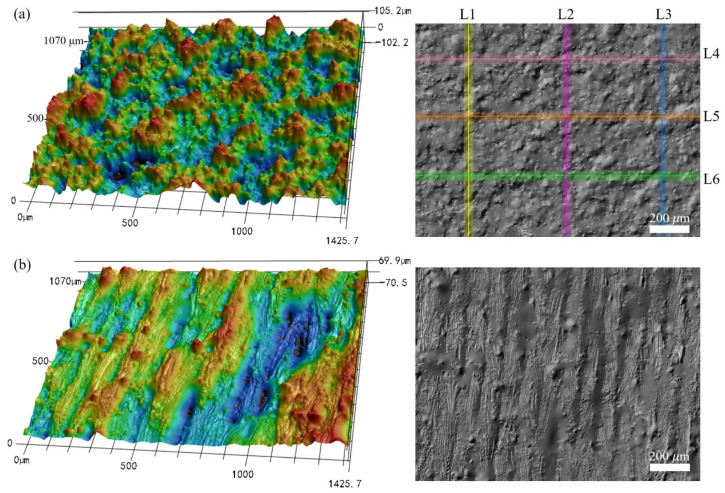
Three-dimensional and two-dimensional surface topographies of the LPBF-built specimen with an inclination angle of 50°: (**a**) the side surface and (**b**) the top surface.

Figure 2 illustrates the 3D and 2D surface morphologies of the LPBF-built sample manufactured with an inclination angle of 50°. As shown in Figure 2a, the peaks or valleys appear non-periodically on the side surface of the LPBF-built samples. The spatial height of the measured surface varies from −102 µm to +105 µm. The morphology of the top surface displays obvious striation patterns along the direction of laser scanning (Figure 2b), and the spatial distribution on the top surface is within the range of −75 µm~+66 µm.

It has been reported that the surface irregularity on the top surface of the fabricated components is mainly attributed to the instability and discontinuity of the laser track caused by the Plateau–Rayleigh capillary instability of the melt pool [21], the waviness formed due to the overlap between adjacent melt tracks [33], as well as the formed laser ripple on the melt track [11]. The multi-track scanning during LPBF leads to partially melted particles or a few adhered powder particles on the top surface of the fabricated component [20,34]. For the side surface, the surface morphology is dominated by the additional angle-dependent surface characteristics involving the staircase effect inherent to the layer-wise process, the partially melted particles, or the serious powder bonding since the down-skin surface is inside the powder bed [35], as well as the agglomeration of peaks due to the insufficient support by the loose and randomly distributed powder particles [18]. Those angle-dependent surface characteristics contribute particularly to the enlargement of the surface irregularity on the side surface.

**Figure 3:**
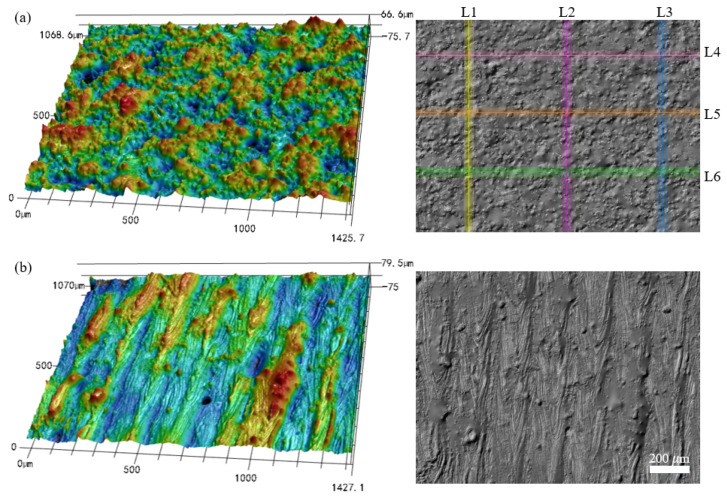
Three-dimensional surface height map of (**a**) the side surface and (**b**) the top surface of the as-built specimen with an inclination angle of 90°.

When increasing the inclination angle to 90°, the height of peaks and depth of valleys on the side surface are both significantly decreased, and the agglomeration of peaks or valleys is improved, as illustrated in Figure 3a. By comparing the surface morphology shown in Figure 2 and Figure 3, it is found that the morphology on the top surface is apparently independent of the inclination angles of the LPBF-built specimen. The explanation is that the surface roughness on the top surface is related to the melting and solidification of the melt pool, which is dominated by energy-related parameters, including the scanning speed, the laser power, and the hatch spacing. At the same time, the inclination angles of the LPBF-built samples significantly affect the surface characteristics on the side surfaces of specimens, which agree well with the findings in existing research [20]. For the down-skin surface of the specimen with a certain inclination angle, the melt pool extension occurs in the powder-supported area [36]. As a consequence, the agglomeration of peaks forms on the side surfaces of the as-built specimen.

**Figure 4:**
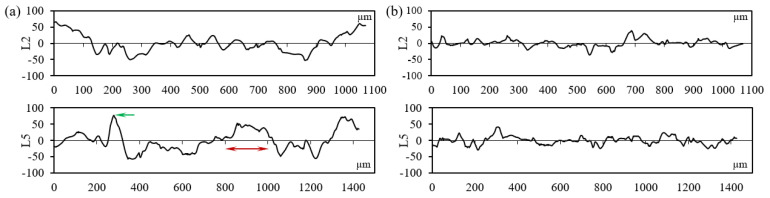
Profile curves on the side surface of LPBF-built specimens with the inclination angle of (**a**) 50° and (**b**) 90°. L2 indicates the profile curve along the vertical direction and L5 is the profile curve along the horizontal direction.

To further evaluate the surface topography of the LPBF-built sample, the profile distribution of the specimen with inclination angles of 50° and 90° is measured and displayed in Figure 4. L2 indicates the profile curve along the vertical direction, while L5 is the profile curve along the horizontal direction, as illustrated in Figure 2. The height of the irregularly shaped peaks achieves 76 µm for the specimen with an inclination angle of 50°, which is pointed out by the green arrow in Figure 4a. The span of those agglomerated peaks achieves 200 µm, as denoted by the red arrow in Figure 4a. Given the powder size distribution (8 µm~50 µm) in this work, considerably large-sized peaks (>50 µm) with irregular shapes are formed mainly due to the melt pool extension attributed to insufficient support from the powder bed, as well as the gathering of the partially melted particles. As shown in Figure 4b, the span of the agglomerated peaks, which corresponds to the powder size distribution, is much smaller, and the height of those peaks is less than 45 µm. Therefore, the formation mechanism of the irregularly shaped peaks on the specimen with an inclination angle of 90° is due to the gathering of partially melted powder particles.

**Figure 5:**
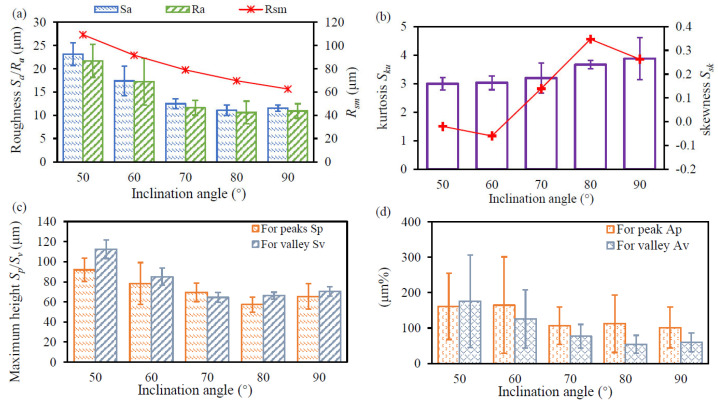
Morphological characteristics of LPBF-built specimens as a function of the inclination angle: (**a**) areal roughness *S_a_* and profile roughness *R_a_*; (**b**) areal kurtosis *S_ku_* and areal skewness *S_sk_*; (**c**) maximum height of peaks *S_p_* and of valleys *S_v_*; and (**d**) cross-sectional area on the 2D profile for peaks *A_p_* and for valleys *A_v_*.

The surface characteristics are summarized in Figure 5. The profile roughness of one measured area is the average value of six profile curves, i.e., L1–L6, shown in Figure 3. As expected, the areal roughness *S_a_* and profile roughness *R_a_* of the down-skin surface decrease with the inclination angle, as shown in Figure 5a. *R_sm_* is introduced to evaluate the staircase effect, and the *R_sm_* model is presented in the reference [20]. The result indicates that the measured surface roughness is dominated by the effect of the staircase. As shown in Figure 5b, the areal kurtosis *S_ku_* equals 3 for the specimen with an inclination angle of 50°, indicating a normal distribution for the height. The *S_ku_* increases slowly to 3.9 when increasing the inclination to 90°, revealing that the height distribution on the measured surface is spiked. The down-skin surfaces on the specimen with an inclination angle of 50° are negatively skewed surfaces, whereas those on the specimens with an inclination angle over 70° are positively skewed. Figure 5c displays the variation in *S_p_* and *S_v_* as a function of the inclination angles. The magnitude of *S_p_* and *S_v_* is decreased when increasing the inclination angle from 50° to 80°, whereas a visible increase can be observed when the inclination angle varies from 80° to 90°. The cross-section of peaks and valleys on the 2D profile is also calculated to identify the predominance of peaks and valleys, as shown in Figure 5d. For the case with an inclination angle of 50°, the cross-sectional area of valleys is higher than that of peaks. However, the peaks occupy the domination of the cross-sectional area if the inclination angle is over 60°.

It was reported that the areal kurtosis *S_ku_* and areal skewness *S_sk_* are discriminating parameters to indicate the comparative predominance of valleys or peaks [20]. In this work, more valleys and fewer peaks are found on down-skin surfaces with negative skewness (the case with an inclination angle of 50°), whereas more peaks and fewer valleys can be seen on down-skin surfaces with positive skewness (the case with an inclination angle over 70°). Additionally, the high value of the kurtosis (>3) also confirms the dominance of peaks on the down-skin surface for the case with an inclination angle over 70°.

### 3.2. Surface Characterizations after Laser Polishing

**Figure 6:**
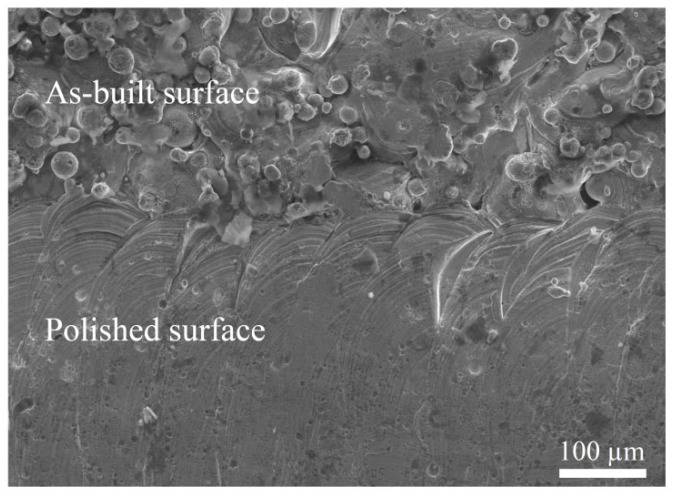
SEM image of the side surface before (**up**) and after (**down**) laser polishing for the specimen with an inclination angle of 50° (P3).

By comparing the surface characteristics between the LPBF-built surface and polished surface in Figure 6, it reveals that performing the laser polishing process could significantly decrease the surface roughness by smoothing the asperities and eliminating the partially melted powder particles, which are attached to the side surfaces of the LPBF-built specimens.

**Figure 7:**
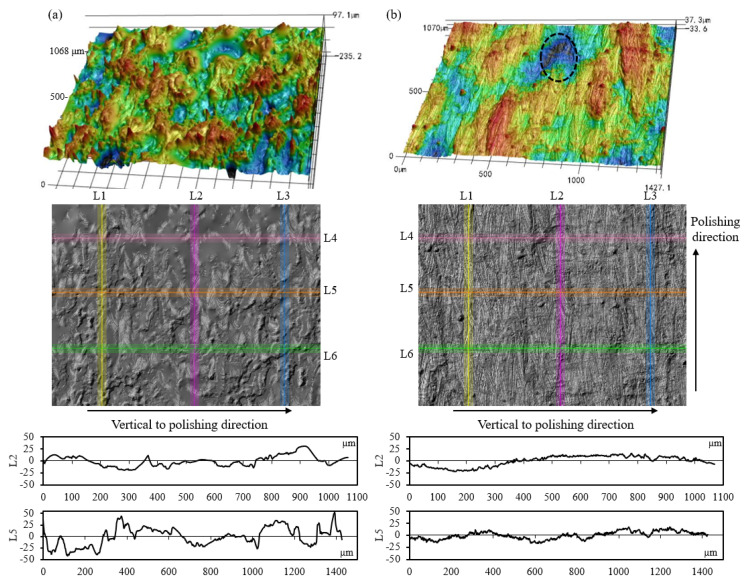
D morphology and 2D profile curve of the polished surface of the specimen with 50° inclination angle: (**a**) 40 W and 300 mm/s (P1), (**b**) 120 W and 300 mm/s (P5).

Figure 7 presents the variation in the surface modifications polished with various laser powers. When using the parameters of 40 W and 300 mm/s (Figure 7a), a high level of irregularity is observed since the original morphological features of the LPBF-built surface are basically preserved. The 2D profile in Figure 7a also reveals that the magnitude of the irregularly shaped peaks with large dimensions is decreased to less than 50 µm. In the case of 120 W and 300 mm/s (P5), the laser polishing can remove the powders that are attached to the as-built specimen surface, as shown in Figure 7b. Highly smoothed profile curves are observed along the laser polishing direction (L2) and the direction vertical to laser polishing (L5). The tiny serrated features on the 2D profiles in Figure 7b represent the laser ripples formed when performing the laser polishing. It has been reported that the formation of those surface ripples mainly comes from the convection within the melt pool at the laser-irradiated zone [37]. It should be pointed out that the deep valleys formed during LPBF (indicated by the black dotted circle in Figure 7b) are still distinguished even if high-power laser polishing is performed.

**Figure 8:**
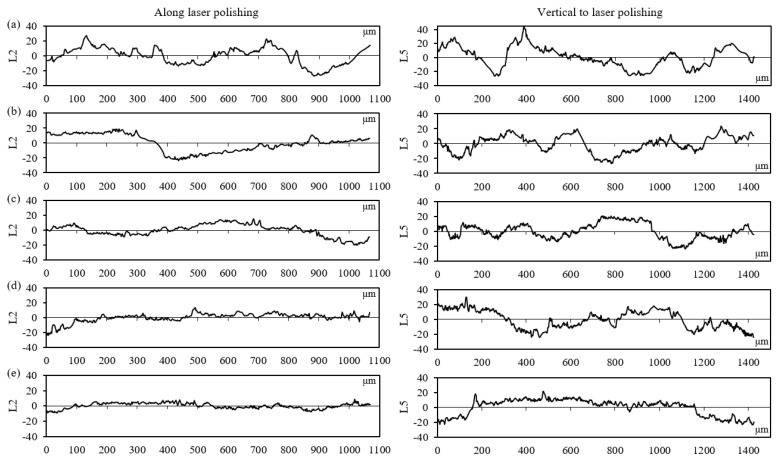
Profile morphology on the polished surface of specimens under various scanning speeds: (**a**) 500 mm/s, (**b**) 400 mm/s, (**c**) 300 mm/s, (**d**) 200 mm/s, and (**e**) 100 mm/s. L2 indicates the profile along the polishing direction, and L5 displays the profile vertically to the polishing direction.

To estimate the influence of the scanning speed on the morphology of the polished surfaces, Figure 8 illustrates the 2D profile curves on the polished surface of the specimen with a 50° inclination angle. The profile morphologies along the laser polishing (L2) and vertical to laser polishing (L5) are all characterized. It is noticeable that a small degree of surface irregularity along the direction of the laser polishing is observed compared with the direction vertical to the laser polishing. Furthermore, the profile morphology is greatly improved when decreasing the laser speed from 500 mm/s to 100 mm/s. When applying the scanning speed of 100 mm/s, the agglomeration of peaks or valleys is eliminated, although noticeable surface waves can still be observed along the direction vertical to laser polishing.

**Figure 9:**
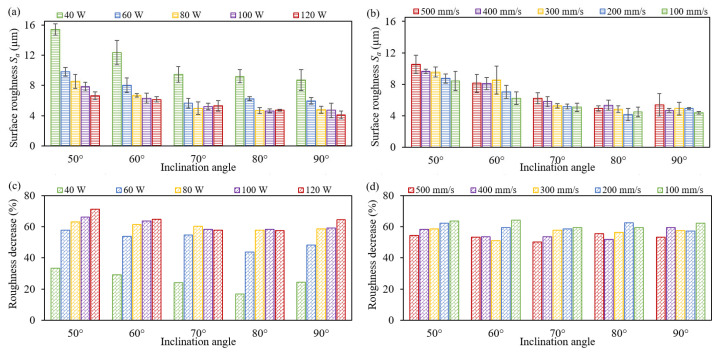
Surface roughness *S_a_* and its decrease after laser polishing: (**a**–**c**) under different laser powers, and (**b**–**d**) under different scanning speeds.

The detailed and qualitative analysis of the polished morphology is performed to reveal the influence of the scanning speed and the laser power on the surface roughness and the roughness reduction after laser polishing, as shown in Figure 9. Under certain polishing parameters, the surface roughness improvement highly relies on the inclination angle of the LPBF-built samples. Figure 9a indicates that the polished surface roughness of the specimen with a 90° inclination angle decreases to 4.09 µm when applying the laser power of 120 W, while it reaches 6.65 µm for the sample with a 50° inclination angle. The reductions of the roughness are 64.52% and 71.29%, respectively, as shown in Figure 9c. For the specimens with the same inclination angle, surface roughness decreases significantly when increasing the laser power from 40 W to 60 W, and a reduction in the roughness becomes slow if keeping to increase the laser power from 60 W to 120 W. Figure 9b shows that the surface morphology can be improved progressively by reducing the scanning speed from 500 mm/s to 100 mm/s.

## 4. Discussion

To further evaluate the influence of polishing parameters as well as the initial surface morphology on the polished morphology, the generalized second-order polynomial response surface model is utilized, and it is defined as shown in Equation (2).

(2) $$Y=\beta_{0}+\sum_{i=1}^{k}\beta_{i}x_{i}+\sum_{i<j}^{k}\beta_{ij}x_{i}x_{j}+\sum_{i=1}^{k}\beta_{ii}x_{i}^{2}\;$$

where *Y* is the expected or predicted value of the dependent variable, $x_{i}$ are the input factors, $\beta_{i}$, $\beta_{ii}$, $\beta_{ij}$ represents the undetermined coefficients, and $\beta_{0}$ is the mean. In this work, the inputs are the initial surface roughness before polishing ($x_{1}$), laser power ($x_{2}$) and scanning speed ($x_{3}$). Here, $x_{i}x_{j}$ means two-factor interaction effects. The output response is the measured surface roughness after laser polishing. Based on the measured data, the Design-Expert program (13.0) was employed for the modeling of the experimental results, and the quadratic polynomial was obtained by choosing the best fitting. The second-order regression model with the polished surface roughness as the predicted value is expressed as the equation below.

(3) $$Y=-2.75246x_{1}-0.744094x_{2}-0.018798x_{3}+{0.029881x}_{1}x_{2}-0.001550x_{1}x_{3}+0.000458x_{2}x_{3}\;+0.050948x_{1}^{2}{+0.001304x}_{2}^{2}{+0.000003983x}_{3}^{2}+53.05046$$

Analysis of variance (ANOVA) plays a crucial role in the response surface model (RSM). The response surface model is a commonly used approximate surrogate model that characterizes the relationship between output variables and input variables by constructing an approximate algebraic model in polynomial function form. In the analysis and optimization process of response surface modeling, analysis of variance is mainly used to test whether different factors (or independent variables) have a significant impact on the response value. In our work, AVONA and the test of significance were used to analyze the model equation, and *p* < 0.05 was taken as the significant term. Table 3 shows the obtained results of the ANOVA. It shows that the initial surface roughness, laser power as well as $x_{1}x_{2}$, $x_{2}x_{3}$, and $x_{2}^{2}$ have a great influence on the surface roughness of polished components. Comparing the *p*-values shown in Table 3, the laser power has the most significant effect on the roughness of the polished surface, followed by initial surface roughness and the scanning speed. The two-factor interaction effect between the laser power and the initial surface roughness is more obvious, while the coefficients of $x_{1}x_{3}$ and $x_{2}x_{3}$ are very large. $x_{2}^{2}$ is also significant terms, while $x_{1}^{2}$ and $x_{3}^{2}$ are non-significant terms. Overall, a model F-value of 18.48 indicates that this model is significant in the series of the experimental parameters used in this work. The predicted *R*^2^ of 0.8061 means that this second-order polynomial surface response model could efficiently predict the polished surface roughness.

**Figure 10:**
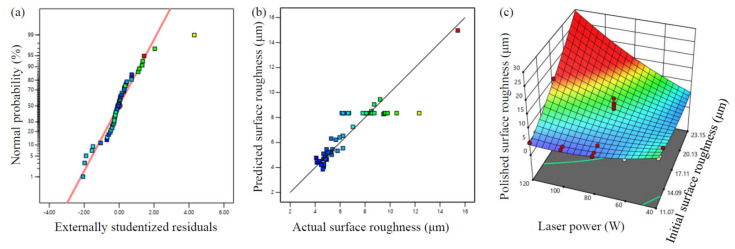
(**a**) Distribution of normal probability with residuals; (**b**) comparison of the actual and predicted values; (**c**) three-dimensional response surface model with the influence of the initial surface roughness and the laser power on the polished surface roughness.

Figure 10a illustrates the residual error normal of our experiment, and nearly all points are beside the diagonal line, indicating a good fit. Figure 10b gives the comparison between the measured surface roughness after laser polishing and the predicted ones. The points in Figure 10b are located on both sides of the diagonal curve evenly. Thus, this regression equation predicts the measured values precisely. The graph of the 3D response surface model for the roughness of the polished surface with regard to the laser power and the initial surface roughness is shown in Figure 10c. With the increasing laser power, the roughness value of the polished surface declines first and then rises. High initial surface roughness, however, results in high polished surface roughness. In the optimization process, the objective function was established to minimize the surface roughness after laser polishing. Based on the regression equation in Equation (3), the optimized processing parameters are obtained by Design-Expert software: the initial surface roughness of the LPBF-built sample is 11.513 µm, the scanning speed is 185.192 mm/s, and the laser power is 110.344 W. In view of the processing accuracy in reality and the capability of the performing equipment, the optimized processing parameters are as follows: the laser power of 110 W, the initial surface roughness of 11.5 µm, and the scanning speed of 185 mm/s. The optimized surface roughness after laser polishing obtained by the prediction model is 2.56 µm, with a roughness reduction of 77.7%.

## 5. Conclusions

In this paper, laser polishing was performed on LPBF-built components, and the identification of the polishing mechanism is dictated by evaluating simultaneously the initial surface morphology prior to laser polishing and the processing parameters during polishing, and our reported methodology can be widely employed towards surface morphology manipulation and process optimization for laser polishing in the additive manufacturing industry. The conclusions are as follows:

(1) For the LPBF-built components, the surface irregularity on the top surface is mainly attributed to the instability and discontinuity of the laser track, the waviness formed due to the overlap between melt tracks, as well as the formed laser ripple on the melt track. While the surface topography of the down-skin surface is dominated by the additional angle-dependent surface characteristics.

(2) For the specimen with an inclination angle of 50°, the irregularly shaped peaks with significantly large dimensions are formed mainly due to the melt pool extension attributed to insufficient support from the powder bed, as well as the gathering of the partially melted particles.

(3) Laser polishing could reduce the surface roughness significantly by smoothening the asperities and eliminating partially melted powder particles attached to the down-skin surfaces of LPBF-built samples.

(4) The profile morphology can be greatly improved when decreasing the laser speed from 500 mm/s to 100 mm/s. With the applied scanning speed of 100 mm/s, the agglomeration of valleys or peaks on the down-skin surface is eliminated, although noticeable surface waves can still be observed along the direction vertical to laser polishing.

(5) One second-order regression model with the polished surface roughness as the predicted value was achieved, and the AVONA results indicate that the laser power has the largest effect on the roughness of the polished surface, followed by the initial surface roughness and scanning speed. The optimized surface roughness after laser polishing of 2.56 µm is obtained by this prediction model, with a 77.7% improvement in surface finish.

As the additive manufacturing industry advances, the demand for improved product surface quality specifications has soared. Therefore, comprehensive research into additional surface polishing techniques and theories is necessary to carry out to fulfill the evolving needs of the industry in the future.

## Figures and Tables

**Table 1:** Processing parameters used to fabricate LPBF-built samples.

Parameters	Value
Laser power *P* (W)	160
Scanning speed *V* (mm/s)	700
Layer thickness *τ* (μm)	30
Hatching distance *H* (mm)	0.1
Laser spot diameter (μm)	60
Rotation angle between layers (°)	47

**Table 2:** Detailed laser polishing parameters.

Case	Laser Power (W)	Scanning Speed (mm/s)	Energy Density (J/mm^2^)
P1	40	300	6.67
P2	60	300	5.56
P3	80	300	4.44
P4	100	300	3.33
P5	120	300	2.22
V1	80	500	2.67
V2	80	400	3.33
V3	80	300	4.44
V4	80	200	6.67
V5	80	100	13.33

**Table 3:** Analysis of variance for the response surface model.

Source	Sum of Squares	df	Mean Square	F-Value	*p*-Value	
Model	217.56	9	24.17	18.48	<0.0001	significant
*x*_1_ initial surface roughness	6.59	1	6.59	5.04	0.0304	
*x*_2_ Laser power	17.32	1	17.32	13.24	0.0008	
*x*_3_ Scanning speed	1.33	1	1.33	1.02	0.3193	
$x_{1}x_{2}$	32.47	1	32.47	24.82	<0.0001	
$x_{1}x_{3}$	2.19	1	2.19	1.67	0.2036	
$x_{2}x_{3}$	6.88	1	6.88	5.26	0.0271	
$x_{1}^{2}$	0.6898	1	0.6898	0.5273	0.4720	
$x_{2}^{2}$	16.60	1	16.60	12.69	0.0010	
$x_{3}^{2}$	0.0968	1	0.0968	0.0740	0.7870	
Residual	52.32	40	1.31			
Lack of Fit	6.69	21	0.3188	0.1327	1.0000	not significant
Pure Error	45.63	19	2.40			
Cor Total	269.88	49				

## Data Availability

The data used to support the findings of this study are available from the corresponding author upon request.
